# Supplementary material for: An ancient lysozyme in placozoans participates in acidic extracellular digestion
Source: Commun Biol. 2026 Jan 6;9:130. doi: 10.1038/s42003-025-09409-6 (PMC12855814; doi:10.1038/s42003-025-09409-6)
Supplement: Supplementary file 1 — Supplementary Information [file 42003_2025_9409_MOESM1_ESM.pdf]

Supplementary Information to

An ancient lysozyme in placozoans participates in acidic extracellular digestion

Henry Berndt[1], Igor Duarte[1], Urska Repnik[2], Michel Struwe[1], Mohammad Abukhalaf[3], Axel Scheidig[1], Andreas Tholey[3], Harald Gruber-Vodicka[1,*] & Matthias Leippe[1,*]

1 Zoological Institute, Kiel University, Kiel, Germany

2 Central Microscopy, Kiel University, Kiel Germany

3 Proteomics & Bioanalytics, Institute for Experimental Medicine, Kiel University, Kiel, Germany

*: Corresponding authors, E-mail addresses: mleippe@zoologie.uni-kiel.de , hgrubervodicka@zoologie.uni-kiel.de

Contents

Supplementary Figures 1 – 14

Supplementary Table 1

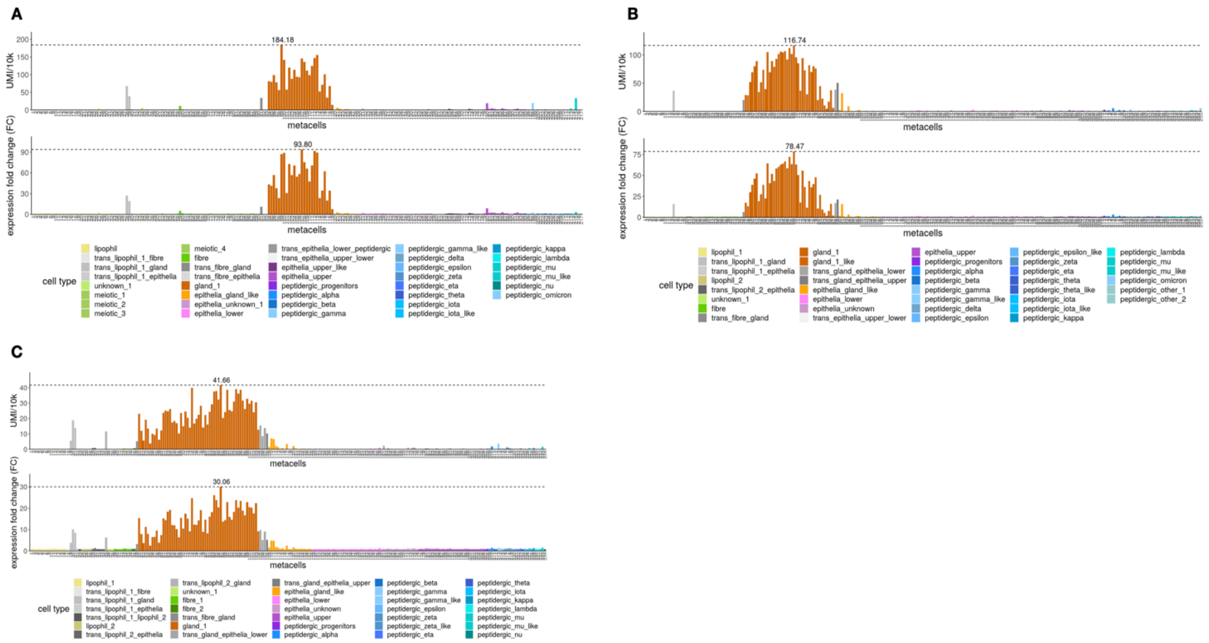

Supplementary figure 1 Expression pattern of *plys* in A) *Trichoplax* sp. H2, B) *Hoilungia hongkongensis* H13, and C) *Cladtertia collaboinventa* H23. The figure was created using the Placozoa Cell Atlas (Najle et al. 2023).

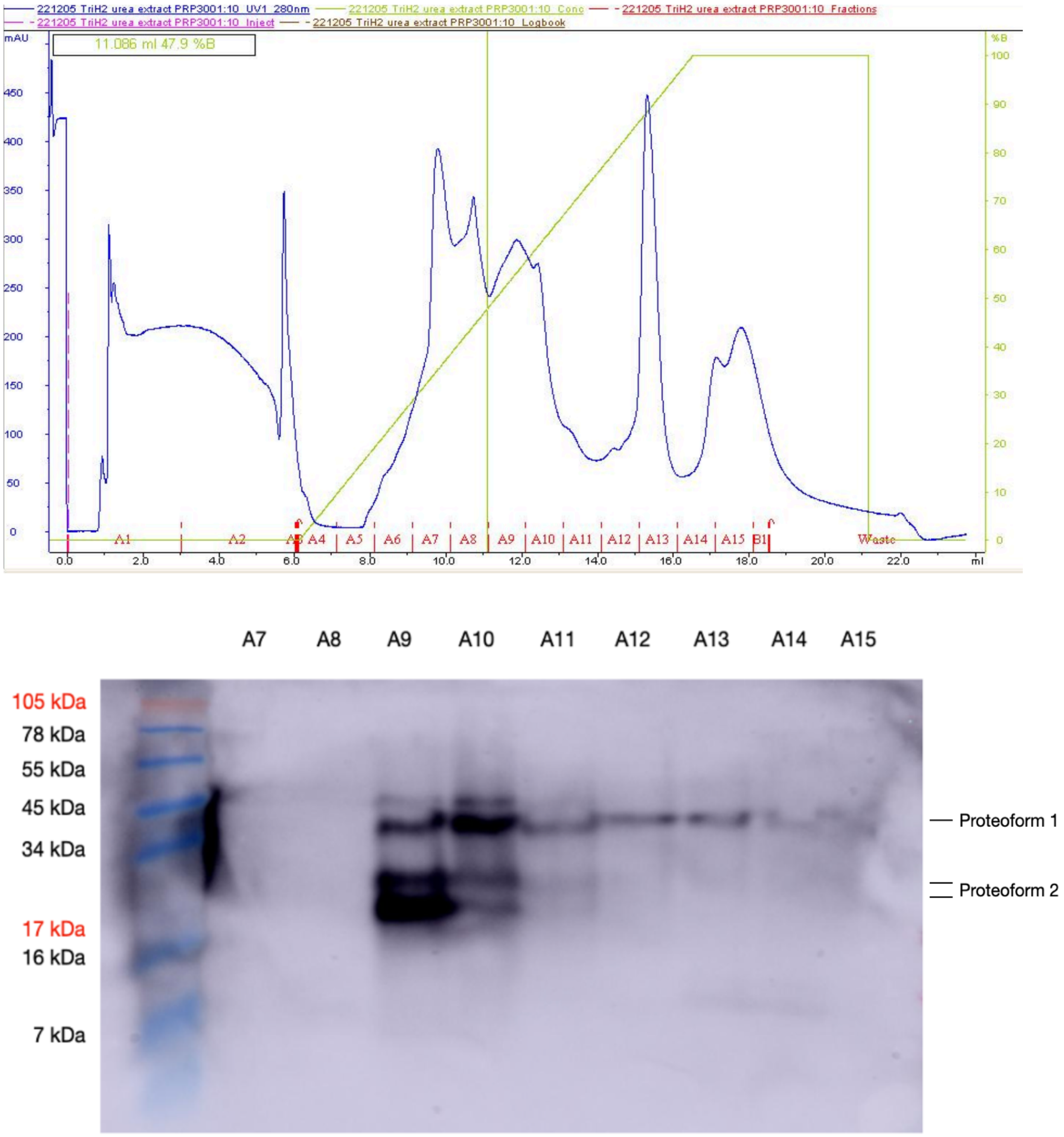

Supplementary figure 2 Chromatogram of the reversed-phase *Trichoplax* sp. H2 protein extract fractionation and Western blot of fractions A7 to A15 with affinity purified anti-PLys IgG. Invitrogen SeeBlue Plus2 standard was used as molecular mass reference for the Western Blot. The two PLys proteoforms are annotated as evidenced by mass spectrometry. Both the chemiluminescence channel and the reflected light channel of the blot acquisition are shown overlaid.

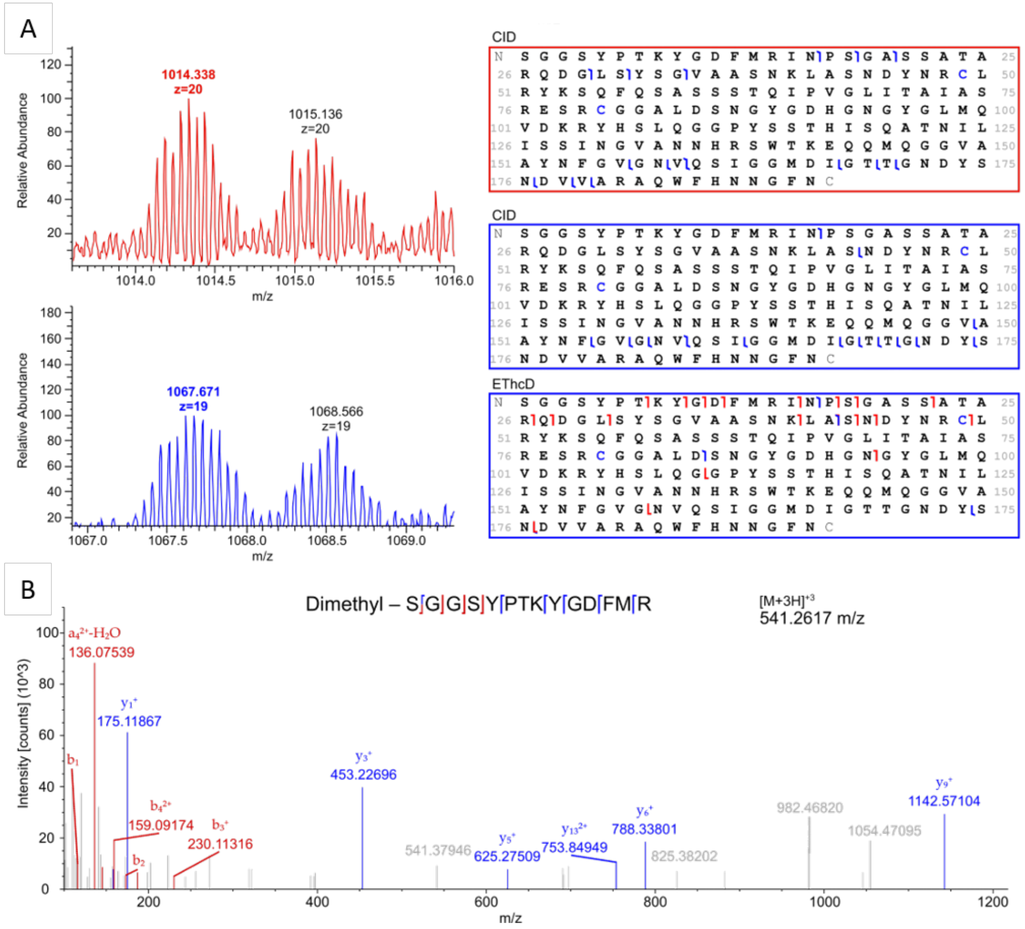

Supplementary figure 3. Representative mass spectrum for the mature Plys (zoom of fig. 1C) acquired in top-down proteomics experiments, and the subsequent sequence information acquired via either CID or EThcD for the charge states z=19 and z=20 (b- y-ions in blue; z- c-ions in red) B) MSMS mass spectrum for the demethylated peptide S^{100}GGSYPTKYGDFMR acquired via bottom-up proteomics based N-terminomics.

Supplementary Table 1 Peptides identified in the bottom-up based terminomics experiment for the mature PLys protein. The neo-terminal peptide starting with Ser100 is highlighted in grey.

Accession	Description	Sum PEP Score	Exp. q-value	Coverage [%]	# Unique Peptides	# PSMs	# AAs	MW [kDa]	Score SequestHT
A0A369RX22	Lysozyme g OS=Trichoplax sp. H2 OX=287889 GN=TrispH2_008933 PE=4 SV=1	67.407	0	50	12	37	290	30.3	31.26

Sequence	Position in Protein	q-value	# PSMs	Theo. MH+ [Da]	Modifications
CGGTTYSGYCPGPSSVR	[076-092]	3.80E-04	1	1805.75814	2×Carbamidomethyl [C1; C10]
CCVSSSGSGGSYPTKYGDFMR	[086-106]	2.40E-04	5	2330.98386	2×Carbamidomethyl [C1; C2]; 1×Dimethyl [K15]
SGGSYPTKYGDFMR	[100-113]	1.50E-03	3	1621.7679	1×Dimethyl [K8]; 1×Dimethyl [N-Term]
INPSGASSATAR	[114-125]	3.80E-04	1	1159.60658	1×Dimethyl [N-Term]
INPSGASSATAR	[114-125]	5.50E-04	4	1131.57528	
QDGLSYSGVAASNKLASNDYNR	[126-147]	2.40E-04	9	2358.13204	1×Dimethyl [K14]
QDGLSYSGVAASNKLASNDYNR	[126-147]	3.80E-04	1	2386.16334	1×Dimethyl [K14]; 1×Dimethyl [N-Term]
SGVAASNKLASNDYNR	[132-147]	3.20E-03	1	1694.84564	1×Dimethyl [K8]
YKSQFQSASSSTQIPVGLITAIASR	[160-184]	2.40E-04	2	2668.43046	1×Dimethyl [K2]
QSASSSTQIPVGLITAIASR	[166-185]	2.40E-04	5	1987.08184	
YHSLQGGPYSSTHISQATN	[204-222]	4.90E-03	2	2047.94681	
NFGVGNVQSIGGMDIGTTGNDYSNDVVAR	[252-280]	2.40E-04	1	2957.36939	
NVQSIGGMDIGTTGNDYSNDVVAR	[257-280]	2.40E-04	1	2499.14162	1×Oxidation [M8]
SIGGMDIGTTGNDYSNDVVAR	[260-280]	2.40E-04	1	2141.97679	

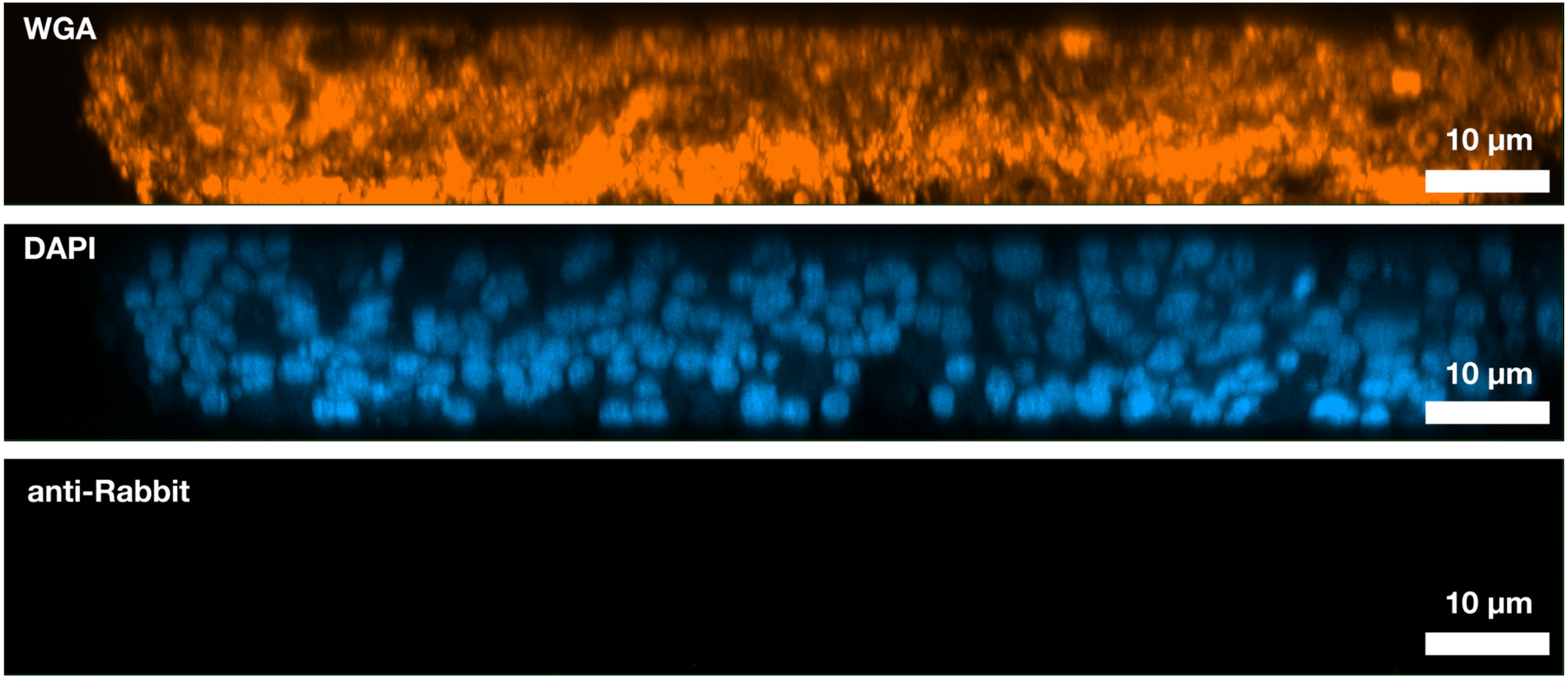

Supplementary figure 4 Orthogonal projections (XZ) of a Z-stack through a wholemount *Trichoplax* sp. H2 after immunohistochemical labeling with 1:100 antiRabbit-FITC conjugate (green), 1:200 WGA-Rhodamine (orange), and 1 µg/mL DAPI (blue). Channels are shown separately in false colors. The orthogonal projections span a depth of 5 µm. Same acquisition parameters were used as for anti-PLys labeling shown in the main text.

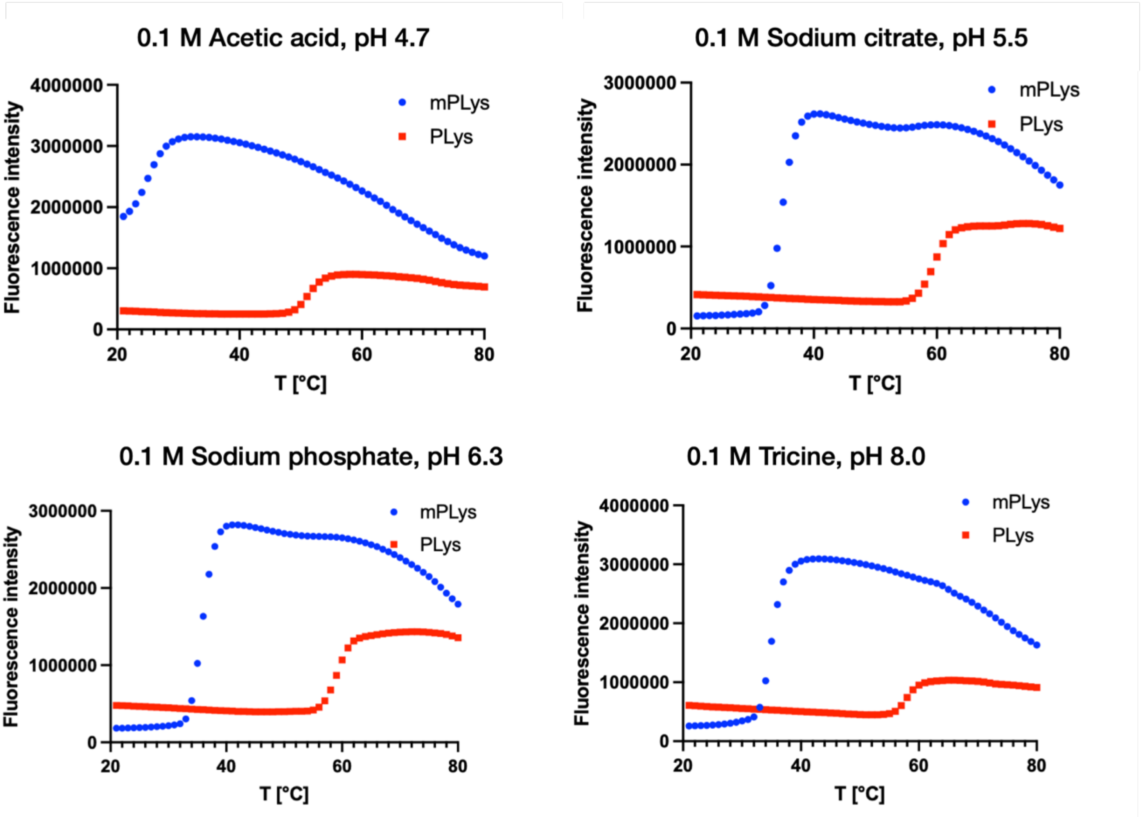

Supplementary figure 5 Representative melting curves of recombinant mPLys_M112 (blue) and pPLys (red) under different buffer conditions.

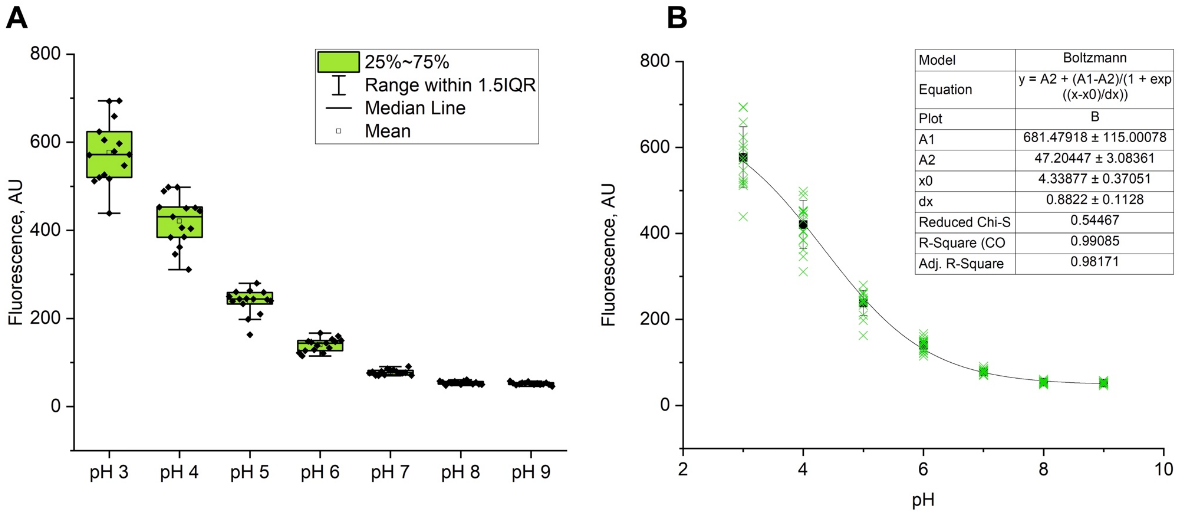

Supplementary figure 6 Calibration curve of pHRodo^{TM} Green zymosan green fluorescence at differing pH values (n=15 per pH). **A**: Boxplot of the data at every pH. **B**: Displayed are the mean values of the data in A as a scatter plot. Individual data points are shown as green crosses. The standard deviation for every mean value is also shown. A sigmoidal function was fitted to the data, parameters of the function and the fitting score are displayed in the legend.

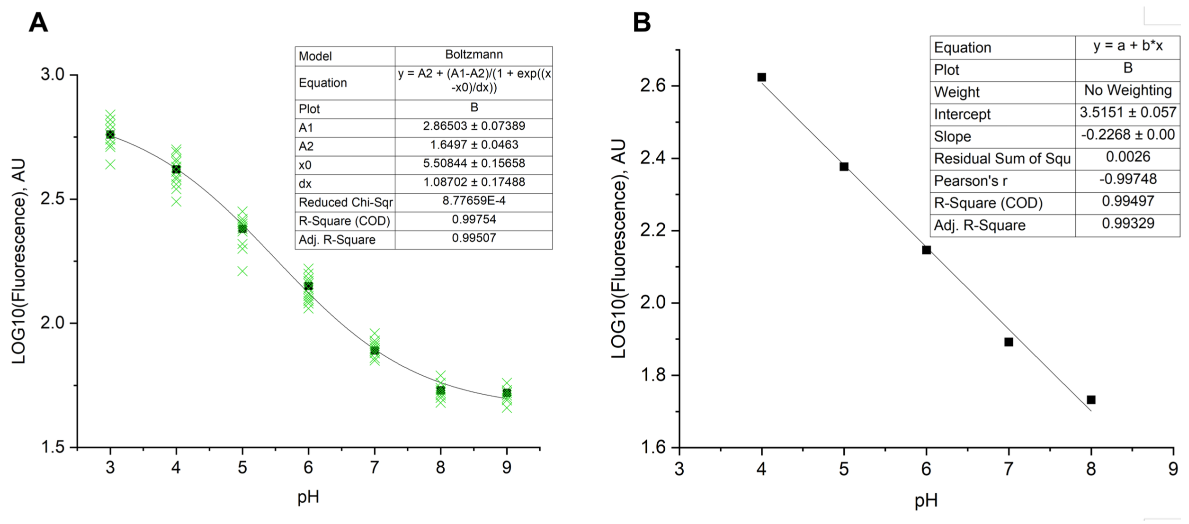

Supplementary figure 7 Calibration curve of pHRodo™ Green zymosan. Shown is the same data displayed in Figure S6 (n=15). **A**: Shown are the logarithmized mean fluorescence values at differing pH values (n=15 per pH). A sigmoidal function was fitted to the data as explained in Figure S6. Individual logarithmized data points are displayed as green crosses. **B**: A linear function was fitted to the mean logarithmized fluorescence values between pH 4 and 8. Parameters of the function and the fitting score are displayed in the legend.

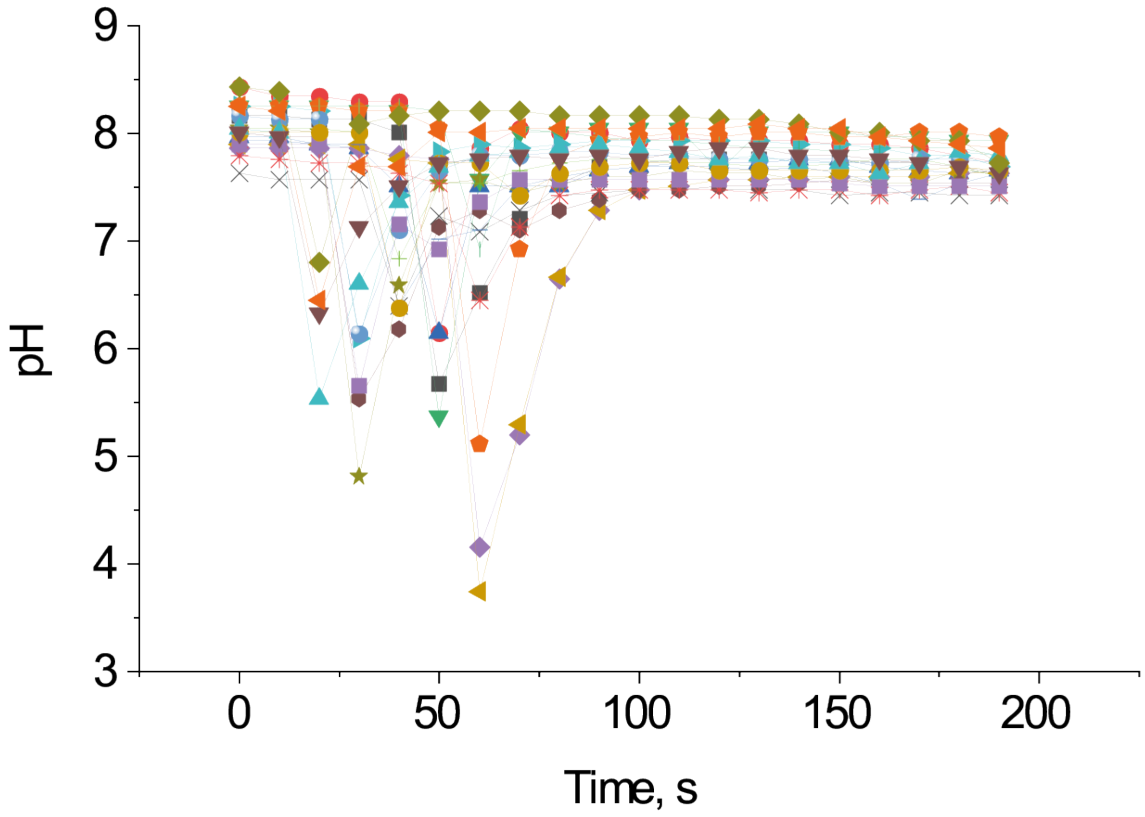

Supplementary figure 8 pH trajectory during extracellular digestive events (n=22) of *Trichoplax* sp. H2. Each feeding event is represented by an individual symbol.

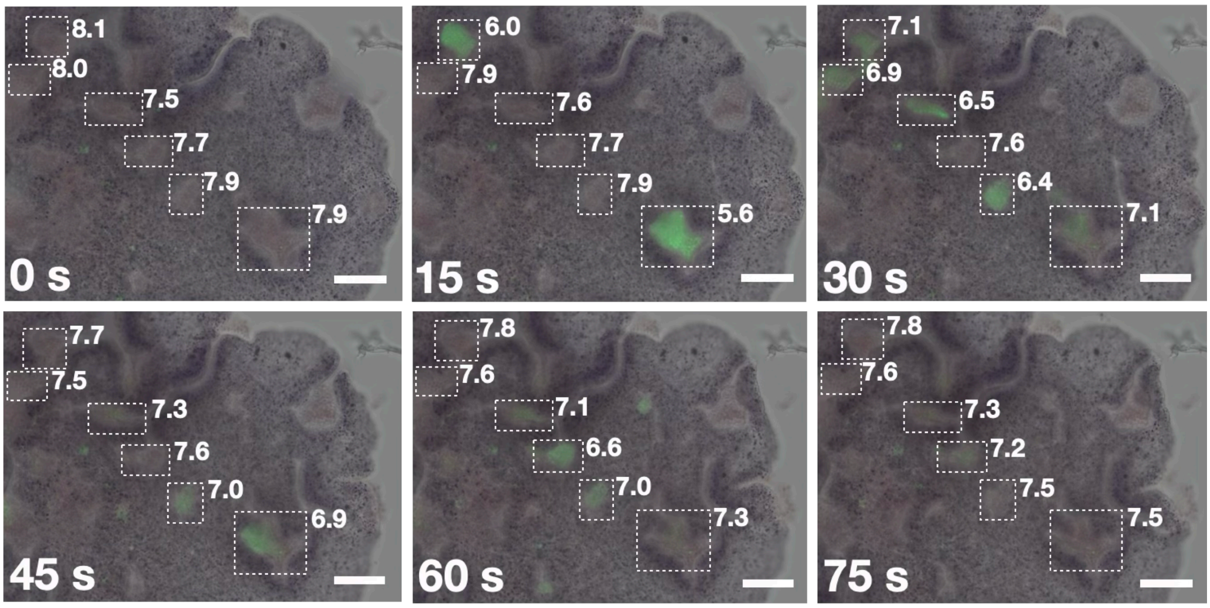

Supplementary figure 9 Merged micrographs in brightfield and green-epifluorescence mode of *Trichoplax* sp. H2 feeding on zymosan particles coated with a pH-sensitive dye at different time points. Feeding grooves are highlighted by dotted lines. The corresponding pH is presented next to the grooves. Scale bars: 50 µm.

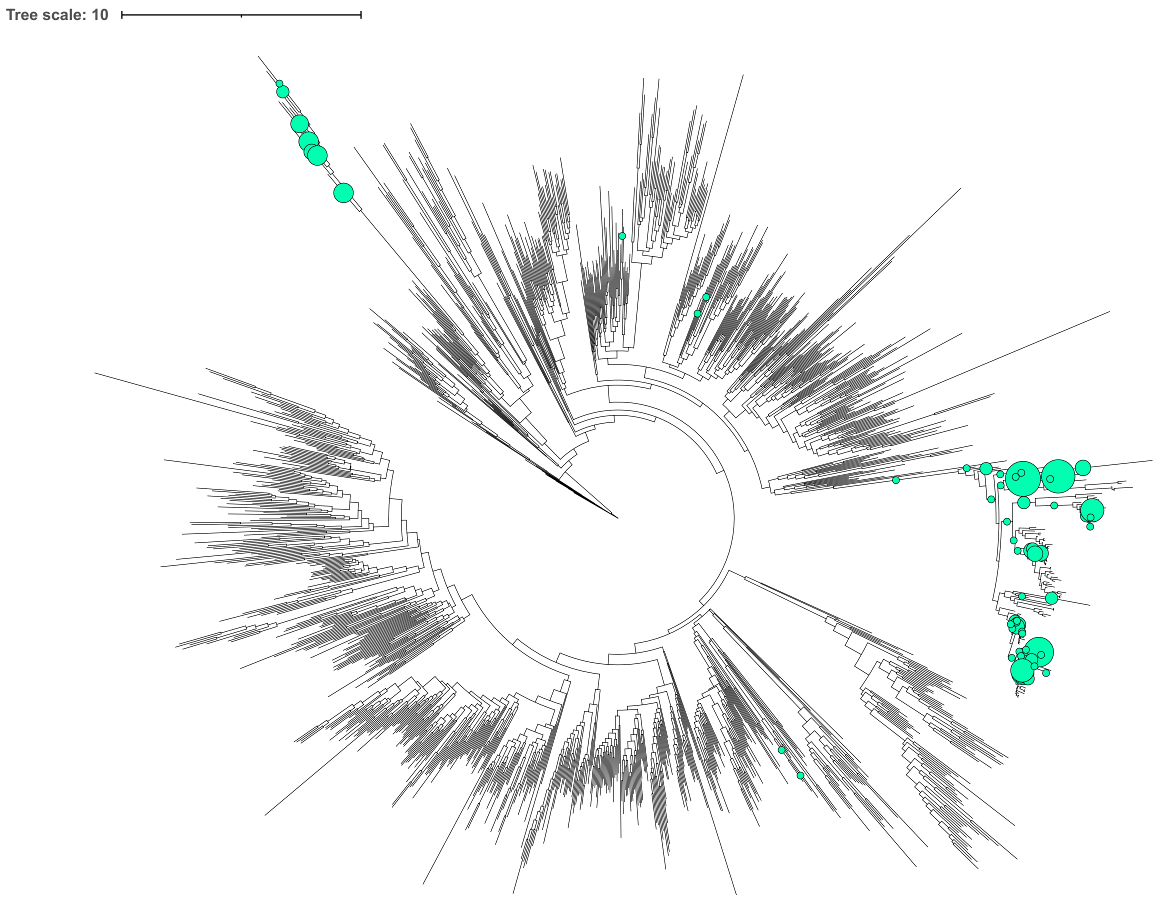

Supplementary figure 10 Phylogenetic tree of glycoside hydrolase family 23 proteins with evolutionary placement analysis results. Circle sizes are proportional to the number of eukaryotic sequences that were placed into a branch following the EPA.

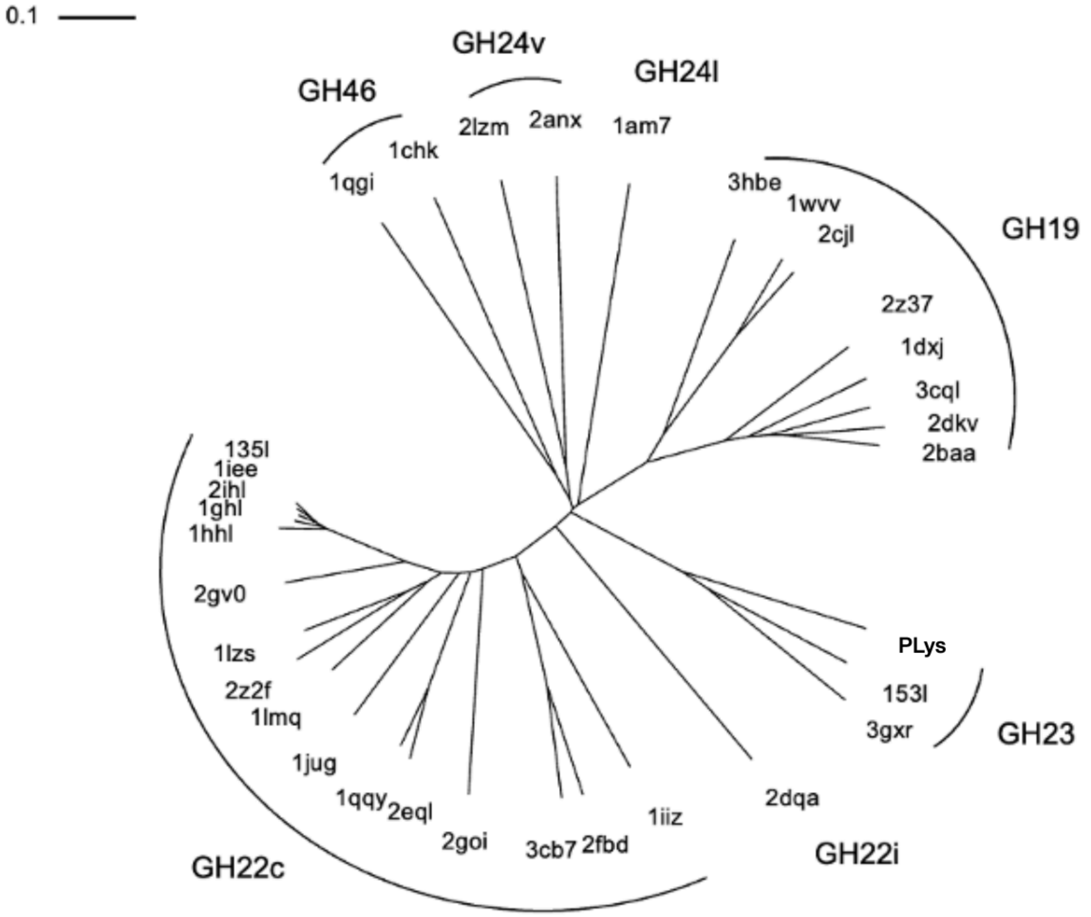

Supplementary figure 11 Structure-based phylogenetic tree of experimentally solved crystal structures of proteins from the lysozyme superfamily and the model of PLys.

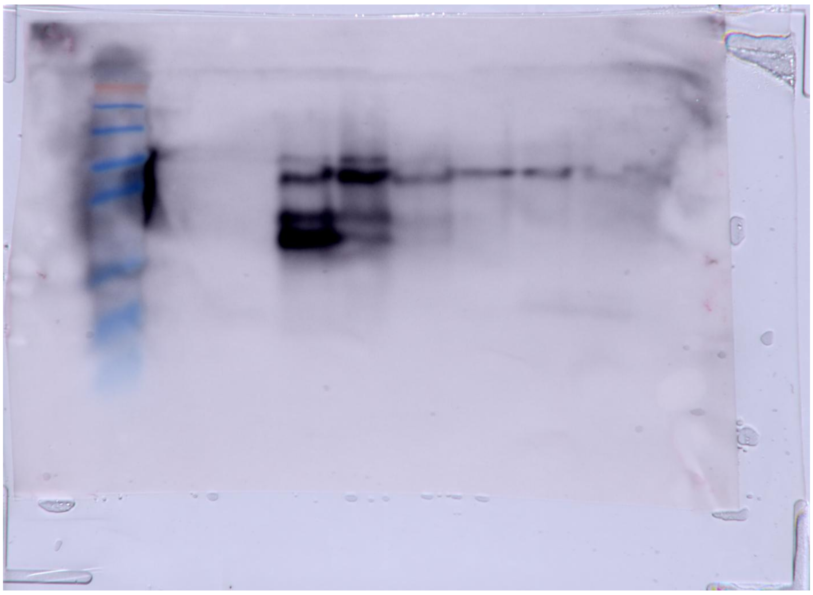

Supplementary figure 12 Raw image of the western blot displayed in Figure S2. Reflected light and chemiluminescence channels were directly superimposed for visualization of marker and ECL signal. The SeeBlue Plus2 protein standard from Invitrogen (Thermo Fisher Scientific) was used.

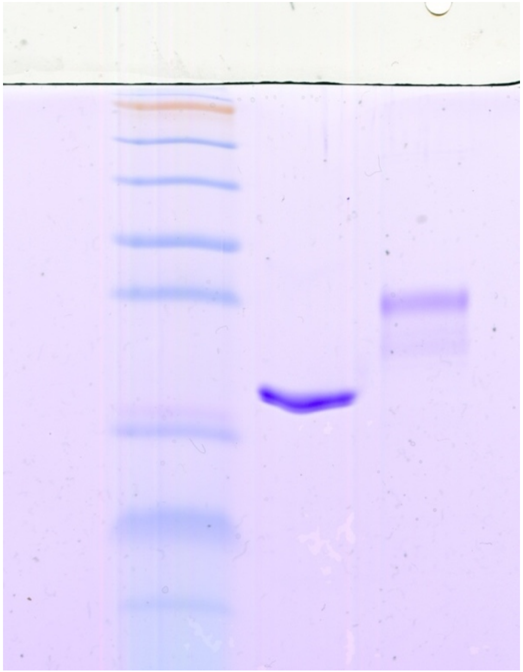

Supplementary figure 13 Raw image of the SDS-PAGE gel displayed in Figure 3. The SeeBlue Plus2 protein standard from Invitrogen (Thermo Fisher Scientific) was used.

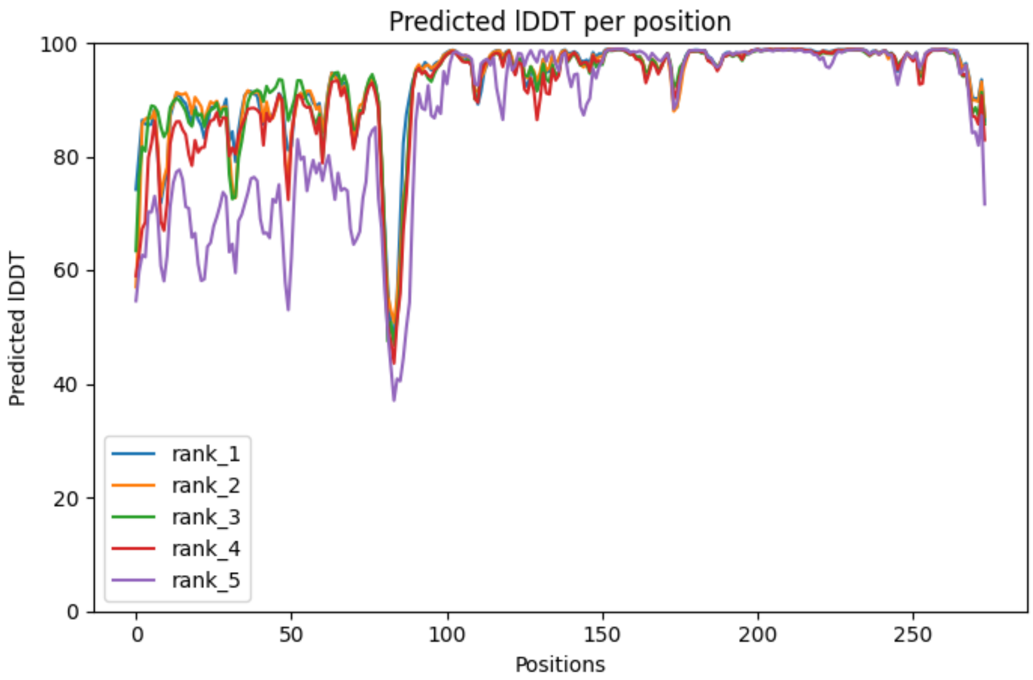

Supplementary figure 14 pLDDT scores for the predicted structure models of PLys with ColabFold v1.5.5. The highest ranked structure was chosen as predicted structure for display and analyses.
