# Supplementary material for: An ancient lysozyme in placozoans participates in acidic extracellular digestion
Source: Commun Biol. 2026 Jan 6;9:130. doi: 10.1038/s42003-025-09409-6 (PMC12855814; doi:10.1038/s42003-025-09409-6)
Supplement: Supplementary file 2 — Description of Additional Supplementary Files [file 42003_2025_9409_MOESM2_ESM.pdf]

Description of Additional Supplementary Files for the manuscript

Supplementary Data 1: Source data for the graphs shown in the manuscript and the Supplementary Material.
